# Hot-melt extruded copper sulfate affects the growth performance, meat quality, and copper bioavailability of broiler chickens

**DOI:** 10.5713/ab.21.0030

**Published:** 2021-06-24

**Authors:** Min Ju Kim, Abdolreza Hosseindoust, Jun Hyung Lee, Kwang Yeoul Kim, Tae Gyun Kim, Byung Jo Chae

**Affiliations:** 1 Centre for Nutrition and Food Sciences, Queensland Alliance for Agriculture and Food Innovation, The University of Queensland, QLD 4072, Australia; 2 College of Animal Life Sciences, Kangwon National University, Chuncheon 24341, Korea; 3 Department of Animal Biosciences, University of Guelph, Guelph, Ontario N1G 2W1, Canada; 4 Darby Genetics Inc., Anseong 17529, Korea; 5 Department of Bio-Health Convergence, Kangwon National University, Chuncheon 24341, Korea

**Keywords:** Bioavailability, Broiler Chickens, Copper Sulfate, Hot Melt Extrusion, Meat Quality, Nano-copper

## Abstract

**Objective:**

This study was conducted to evaluate the effects of the supplementation of diets of broiler chickens with hot-melt extruded CuSO_4_ (HME-Cu) on their growth performance, nutrient digestibility, gut microbiota, small intestinal morphology, meat quality, and copper (Cu) bioavailability.

**Methods:**

A total of 225 broilers (Ross 308), one-day old and initial weight 39.14 g, were weighed and distributed between 15 cages (15 birds per cage) in a completely randomized experimental design with 3 treatments (diets) and 5 replicates per treatment. Cages were allotted to three treatments including control (without supplemental Cu), IN-Cu (16 mg/kg of CuSO_4_), and HME-Cu (16 mg/kg of HME processed CuSO_4_).

**Results:**

The HME-Cu treatment tended to increase the overall body weight gain (p<0.10). The apparent digestibility of Cu was increased by supplementation of HME-Cu at phase 2 (p<0.05). The *Escherichia coli* count in cecum tended to decrease with the supplementation with Cu (p<0.10). In addition, the HME-Cu treatment had a higher pH of breast meat than the control and IN-Cu treatments (p<0.05). Significant increases in the cooking loss, water-holding capacity, and lightness in the breast were observed in the HME-Cu treatment compared to the control (p<0.05). The Cu content of excreta increased with the Cu supplementation (p<0.05). The concentration of excreta Cu in broilers was decreased in the HME-Cu compared to the IN-Cu in phase 2 (p<0.05). The Cu concentration in the liver was increased with the HME-Cu supplementation, compared with the control diets (p<0.05).

**Conclusion:**

This study showed that HME-Cu supplementation at the requirement level (16 mg/kg diets) in broiler diets did not affect the growth performance and the physiological function of Cu in broilers. However, supplementation of Cu in HME form improved the meat quality and the bioavailability of Cu.

## INTRODUCTION

Cu is a fundamental trace element for the growth and metabolic function of broilers, which is involved in haematopoiesis, bone development, and production of enzymes [1,2]. Supplemental Cu in diets has been shown to minimize the adverse effect of reactive oxygen species by suppressing the peroxidation of lipids, thus affecting the nutritive value and meat quality in chickens and turkeys [3,4]. The nutrient requirements of poultry [5] suggested a Cu requirement of 8 mg/kg for normal body functioning in broiler chickens. Cu requirement in broiler chickens was increased to 16 mg/kg, as suggested by Aviagen [6]. However, the requirement level of Cu level in broilers has shown diverse effects on the growth performance and physiological function of Cu, as Cu absorption is dependent on the Cu source [7–10]. CuSO_4_ as traditional Cu additives in industry practice has high solubility that releases reactive free Cu ions quickly in upper gastrointestinal track, which is likely to cause Cu ions to combine with anti-nutrients [11,12]. Thus, CuSO_4_ showed relatively low absorption and bioavailability [2,13].

Nano-Cu that has the higher active surface area has been considered as an alternative to CuSO_4_, increasing the metabolic functions and bioavailability of Cu in chickens [14–17]. However, nano-sized mineral particles also tend to aggregate among themselves, which interrupt the absorption by increasing particle size to micro [18]. Hence, our knowledge suggests that it needs to be processed in CuSO_4_ by the HME technique along with biopolymers preventing the aggregation of Cu ions to increase Cu absorption and bioavailability.

The hot-melt extrusion (HME) is a technology that has been widely used in drug delivery systems, which can regulate the release of functional materials such as pharmaceutical drugs to improve the availability of them by forming dispersion in the gastrointestinal system [19,20]. Previous studies showed that HME-processed trace elements had a reduced particle size of nano by the addition of pharmaceutical excipients to increase the solubility and dispersion of trace elements [21,22]. Our previous studies have shown the improved bioavailability and metabolic function of HME-processed trace minerals in weanling pigs and broilers [23–28]. To evaluate the extent of the nano-process effect on minerals bioavailability in broiler chickens many studies are required, particularly that there are insufficient reports about these technologies at present. The HME process was applied to produce nano size Cu to increase the bioavailability of Cu. Therefore, this study was conducted to investigate the effects of the supplementation of nano-Cu processed using the HME technology in broiler diets by evaluating growth performance, nutrient digestibility, gut microbiota, small intestinal morphology, meat quality, and Cu bioavailability.

## MATERIALS AND METHODS

### Preparation of HME-Cu

The HME-Cu was prepared using the methods described in the study of Koo et al [22]. It resulted in characteristics equivalent to those achieved in the study of Koo et al [22]. Briefly, the premix of CuSO_4_ with polymers (Span 80, Tween 80, and polyethylene glycol [PEG] 6000) was conducted as a 20:12:4:64 ratio to increase the uniformity prior to the HME process. The processed mixture was moved to a feed hopper at a speed of 45 g/min. A hot-melt extruder (STS-25HS; Hankook E.M. Ltd., Pyeongtaek, Korea) that comprised a twin-screw system was used to process the CuSO_4_. The temperatures of the barrel and die sections (with a 1-mm diameter) were sustained at 45°C and 40°C, respectively. The mixture passed through the twin-screw barrel under constant pressure and temperature, and the processed kneading was disconnected with a cutter at the end of the die section. The extruded substances were cooled and milled with the HBL-3500S grinder (Samyang Electronics Co., Gunpo, Korea).

### Experimental design and bird management

This study was conducted at the poultry research facility of Kangwon National University in Chuncheon, Republic of Korea and was approved by the University’s Institutional Animal Care and Use Committee (Approval No: KW-180907-1). A total of 225 birds (Ross 308, one day old) were used as the experimental animals, and the initial body weight (BW) of the birds averaged 39.14±0.97 g. The chicks were randomly put into the cages that were each equipped with a hopper feeder and five water nipples, assigned in the three experimental diets consisting of: Control (without supplemental Cu), IN-Cu (supplemental CuSO_4_, 16 mg/kg diets), HME-Cu (supplemental HME processed CuSO_4_, 16 mg/kg diets). This experiment was conducted in a completely randomized design (CRD), with 3 treatments and 5 replicates of 15 birds per replicate. This trial was performed for 35 days by feeding the birds with diets according to their growth phases (phase 1, days 0 to 21 and phase 2, days 22 to 35). The nutrient contents of the experimental diets (Table 1) were met or exceeded by Aviagen [6], and the dietary Cu level in treatments was shown in Table 2. The birds were raised in floor pens (1.1×1.6 m) covered with rice husks (about 5 cm high) and were allowed to take the feed and water *ad libitum*. The chicks were kept at a temperature of 34°C from days 1 to 7, and thereafter, the room temperature was gradually decreased to 3°C each week until it reached 24°C. All the birds were kept under continuous lighting for 24 hours a day for the first day and then gradually reducing to 16 hours of light and 8 hours of dark during on day 35.

### Growth performance and sampling

The BW and feed intake (FI) of individual birds per pen were recorded at the start and end of each phase to determine their body weight gain (BWG) and gain to feed ratio (G:F). On days 14 and 35, five chicks per replicate were chosen for the collection of blood samples. The blood samples were taken from the wing vein and kept in heparin sodium tubes and centrifuged at 3,000 rpm for 15 minutes at 4°C for the collection of plasma samples. The samples were stored at −80°C as required for analysis [29]. To get tissue samples, five birds per replicate were randomly selected based on average weight of each replicate and slaughtered at the end of the trial, and tissue samples were collected from their liver, small intestine, and muscle (breast and thigh). The liver tissue samples were rinsed with phosphate buffered saline (PBS) with a pH of 7.4 to remove any red blood cells and clots, and were stored at −80°C for analysis of their Cu contents. The small intestines were laid out on a plastic surface and three 5 cm segments were taken from the duodenum (taken at the midpoint of duodenum), jejunum (taken from between bile duct and Meckel’s diverticulum), and ileum (taken from anterior of ileo-cecal junction). The small intestine samples were washed by phosphate buffer solution and stored in a 10% neutral buffered formalin solution (pH 7.2 to 7.4) until required for analysis [8]. After collecting the samples in the small intestine, the excreta in the entire small intestinal tract and cecum were collected. Then the samples were moved immediately to the laboratory for analysis of their microbiota. The muscle samples were kept at 2°C to 4°C for 24 hours [30].

### Nutrient digestibility

Three birds per pen were transferred to metabolic cage to conduct the trial in nutrient digestibility. The chickens were fed diets with chromic oxide (0.25%) added for the last 10 days (adaptive and collective periods) of each phase to determine the apparent total tract digestibility of dry matter (DM), gross energy (GE), crude protein (CP) and Cu. The excreta samples per replicate were collected into stainless steel trays and then dried in a forced-air oven at 60°C for 72 hours, grounded in a Wiley Mill (Thomas Model 4 Wiley Mill; Thomas Scientific, Swedesboro, NJ, USA) using a 1-mm screen, and used for chemical analysis. Each sample was analyzed in triplicate for DM (Method 930.15) and CP (Method 990.03), according to the methods of AOAC [31]. The Cu in the feed, and excreta was determined by inductively coupled plasma emission spectroscopy (ICP) according to the methods of AOAC [31]. The GE of the diets and excreta were measured using a bomb calorimeter (Model 1261, Parr Instrument, Molin, IL, USA), and the chromium concentrations were determined with an automated spectrophotometer (Shimadzu, Japan) according to the procedure described by Fenton and Fenton [32].

### Gut microbiota

The excreta samples from the small intestine and the cecum per replicate were pooled in sterilized plastic bottles and mixed. The samples (1 g) were put together in anaerobic dilution solution (9 mL) by keeping them under CO_2_. The serial dilutions (10^−5^ to 10^−8^) were made by using the anaerobic dilution solution and diluted solution and were plated at 1 mL onto agar plates in triplicate. The total anaerobic bacteria were determined on plate count agar (Difco Laboratories, Detroit, MI, USA) and incubated at 37°C for 24 hours under anaerobic condition. The *lactobacillus* spp. was determined on MRS agar (with 0.20 g/L NaN_3_ + 0.50 g/L L-cystine hydrochloride monohydrate) and incubated at 39°C for 48 hours under anaerobic condition. The *Clostridium* spp. was determined on tryptose sulphite cycloserine agar (Oxoid, Hampshire, UK) and incubated at 37°C for 24 hours under an anaerobic condition. The *Escherichia coli* (*E. coli*) was determined on MacConkey agar and incubated at 37°C for 24 hours under anaerobic condition. The anaerobic condition was made in a gas-pack anaerobic system (BBL, No. 260678; Difco, USA) [33].

### Small intestinal morphology

The duodenum, jejunum, and ileum samples were processed with ethanol for a day and then embedded in standard paraffin. The intestinal samples were stained with azure A and eosin and then crypt-villus samples (total of 10 intact) were selected for the intestinal cross-sections. The samples were measured in triplicates using an optical microscope and an image analysis system (Media Cyber Genetics, Optimus software version 6.5, North Reading, MA, USA). The villus height (VH, μm) was determined from the top of the villus to the crypts of Lieberkühn, and the crypt depth (CD, μm) was measured as the depth of the invagination between the adjacent villi. All the morphological characteristics were analyzed in 10-μm increments.

### Meat quality analysis

About 10 g of the breast and thigh muscles were excised and homogenized with distilled water (90 mL) at 10,000 rpm for 8 minutes. The pH values of the breast and thigh muscles were determined using a precise pH meter (ST2100-E; OHAUS, Parsippany, NJ, USA) [34]. The cooking loss (CL) was determined according to Sifa et al [30] procedure. The meat samples (about 10 g) were weighed and placed into plastic bags and then cooked at 80°C for 30 minutes in a constant-temperature water bath. Then the samples were kept at room temperature to be cooled and reweighed. The CL of muscle was calculated as follows:


CL (%)=[(initial weight-cooked weight)/initial weight]×100

The water-holding capacity (WHC) was estimated through the method reported by Lee et al [35]. The meat samples (approximately 1.0 g) were placed between 18 pieces of 1 cm-diameter filter paper and then pressed at 35 kg for 6 minutes. The amount of expressed juice was defined as the weight loss that occurred after pressing and was presented as a percentage of the initial sample weight. The WHC was calculated as follows:


WHC (%)=[(initial weight-weight after pressing)/initial weight]×100

The meat color was estimated with a chromameter (ChromaMeter CR-300; Minolta Co. Ltd., City, Japan). The average of the triplicate measurements was recorded, and the result was expressed as the International Commission on Illumination (CIE) LAB values of lightness (L*), redness (a*) and yellowness (b*) [30].

### Cu contents

The Cu contents of the feed, excreta, plasma, and liver were determined by ICP according to the methods of AOAC [31]. Briefly, the ground feed and fecal samples (about 1 g) were weighed and heat-treated at 600°C for 1 hour in an electric muffle oven. The ashed samples then were cooled and lysed with 10 mL of 50% HCl (v/v) and were kept covered overnight. The samples were filtered using Whatman filter paper into a 100 mL flask known as a volumetric flask, which was washed two to three times, after which the samples were diluted with deionized distilled water and their Cu concentrations were measured with ICP. For the plasma samples, 1 mL samples were measured in porcelain crucibles and oven-dried for 4 hours at 105°C and then ashed for 1 h at 600°C in a muffle furnace. Liver samples were milled with a blade grinder after dried out for 24 hours at 105°C. The sample (1 g) was estimated and heat-treated at about 600°C for 1 hour in a muffle furnace. The heat-treated plasma and liver were dissolved by adding to 10 mL of 50% HCl (v/v) and were kept covered overnight. For removing floating matter from the samples, the dissolved solution was filtered using a Whatman filter into a measuring flask washed two to three times, after which they were adulterated with de-ionized distilled water and their Cu concentrations were detected with ICP.

### Statistical analysis

All the data were evaluated by performing one-way analysis of variance procedure of SAS (SAS Inst. Inc., Cary, NC, USA) [36] in CRD. Significant statistical differences among the treatment means were determined using Tukey’s Honestly Significant Difference test. Pens were considered the experimental units for growth performance and nutrient digestibility, and birds, the experimental units for gut microbiota, small intestinal morphology, meat quality, and Cu contents. Statistical significance was set at p<0.05, and <0.10 was considered a trend towards significance.

## RESULTS

### Growth performance

The growth performance results are shown in Table 3. The supplemental Cu had no significant effect on the BWG, FI, and G:F in phase 1. However, the 16 mg/kg supplemental HME-Cu in phase 2 tended to increase the BWG in phase 2 (p<0.10).

### Nutrient digestibility

The nutrient digestibility results are shown in Table 4. There was no significant difference between the treatments. Supplementation of HME-Cu significantly increased the Cu digestibility compared to control and IN-Cu at phase 2 (p< 0.05).

### Gut microbiota

The gut microbiota results are shown in Table 5. The supplemental Cu had no effect on the population of total anaerobic bacteria, *Lactobacillus* spp., *Clostridium* spp., and *E. coli* spp. in the small intestine. However, the *E. coli* count in the cecum tended to decrease with the Cu supplementation (p<0.10).

### Small intestinal morphology

The small intestinal morphology results are presented in Table 6. There was no significant difference with the Cu supplementation.

### Meat quality

The meat quality results are shown in Table 7. The supplemental Cu significantly decreased the pH values in the breast and thigh compared to the control (p<0.05). In addition, the HME-Cu treatment had a lower pH than the IN-Cu treatment (p<0.05). However, significant increases in the CL, WHC, and lightness in the breast were shown not only with the Cu supplementation compared to the control, but also with the HEM-Cu supplementation compared to the IN-Cu (p<0.05). The supplemental HME-Cu significantly increased the CL, WHC, and lightness compared to the control (p<0.05).

### Cu bioavailability

The resulting Cu concentrations in the plasma, liver, and excreta are presented in Table 8. There were no significant differences in the plasma between the supplemental Cu treatments and the control in phases 1 and 2. The Cu content in the excreta increased with the Cu supplementation in phases 1 and 2 (p<0.05). In addition, the HME-Cu supplementation decreased the Cu content in the excreta compared to the IN-Cu supplementation in phase 2 (p<0.05). The Cu concentration in the liver significantly increased in the chickens fed diets supplemented with HME-Cu compared to the chickens fed control diets (p<0.05).

## DISCUSSION

In chickens, Cu sources that improve Cu bioavailability, such as amino acid chelated Cu, tribasic copper chloride (TBCC), copper-loaded chitosan, and nano-Cu, have been considered alternatives to inorganic Cu [37]. Nano-Cu increases Cu bioavailability in chickens, and only small doses of it stimulate biochemical and physiological processes, because nanoparticle minerals have the distinct feature that is similar to the increased active surface area of colloid [20]. In this study, 16 mg/kg of supplemental Cu in both IN-Cu and HME-Cu tended to increase the BWG at phase 2. In accordance with previous study, supplementation of 15 mg/kg Cu, CuSO_4_, or Cu_2_O, had tendance increasing the average daily gain of broilers [38]. In addition, Wang et al [39] reported that 50 mg/kg nano-Cu supplementation in diets increased the BWG in broiler chickens.

The supplementation with Cu (16 mg/kg) and the Cu sources did not affect the nutrient digestibility such as DM, GE, and CP in this study. As the similar result from this study, Lee et al [40] reported that no significant effect of nutrient digestibility in broilers fed diet that inorganic or nano-Cu was supplemented with gradual increase of Cu level (16 to 120 mg/kg) was induced. In contrast with this result, supplementation with 50 mg/kg of nano-Cu increased the energy and fat digestibility compared to the control diets in pigs [41]. Dietary supplemental Cu increases the enzyme activity such as pancreatic lipase and phospholipase A, which is concerned with Cu homeostasis. The exceeded tolerance of Cu in liver is released through pancreatic duct with pancreatic juice that contains digestible enzymes [42]. These results in this study mean that 16 mg/kg supplemental Cu might be not enough to improve in DM, GE, and CP digestibility. However, the increased Cu digestibility was induced by HME-Cu in this study. In agreement with this data, Xiangdi et al [43] reported that 8 mg/kg of supplemental nano-Cu increased the apparent digestibility of Cu compared to CuSO_4_.

This study showed that supplementation with 16 mg/kg of Cu tended to decrease *E. coli* population in cecum. In contrast, 150 mg/kg of CuSO_4_ in diets decreased the abundance of Streptococcaceae and Corynebacteriaceae, Gram-positive bacteria, in ileum [38]. Furthermore, Wang et al [39] reported that supplementation with 100 mg/kg of copper-loaded chitosan nanoparticles in broiler diets not only increased the *Lactobacillus* and *Bifidobacterium* populations in cecum but also decreased the *E. coli* population compared to the control. Nano-sized Cu displays noticeable antimicrobial activity due to it’s role as antibacterial surface agent that provokes membrane damage with oxidative stress and DNA degradation to hazardous bacteria [44]. The significant antibacterial effect of Cu in intestine would be induced with dietary high concentration of Cu (100 to 250 mg/kg diet) [45].

In the small intestinal morphology, there was no significant effect of supplemental Cu in this study. The similar result with this study was indicated by Xia et al [46] who reported that the supplementation with 36.75 mg/kg of CuSO_4_ had no effect on the VH and CD of small intestine in broilers. However, Attia et al [47] showed that the intestinal mucosa of the ileum was affected by administrations with the 120 mg/kg of Cu in hens.

The meat quality is influenced by pH, WHC, and CL. In particular, the pH value in meat reflects the muscle acid status and affects the WHC, CL, and color [35,48]. The normal pH in chicken meat is known to be 5.7 and 6.1. Chicken meat with a pH below 5.7 and above 6.1 is referred to as acid and DFD (dark, firm, and dry) meat, respectively [49]. In this study, the breast and thigh fillets of the broilers fed diets supplemented with 16 mg/kg of Cu had normal pH values, unlike those of the broilers fed the control diets. The data are consistent with those of Winiarska-Mieczan and Kwiecień [50], who reported that 16 mg/kg dietary supplemental Cu showed a decline in the pH of the breast and thigh meat of chickens compared to 8 mg/kg dietary supplemental Cu. In addition, dietary supplementation with HME-Cu decreased the pH and increased the WHC of the breast and thigh fillets compared to the control diets or dietary supplementation with IN-Cu. The WHC is associated with the pH and a higher WHC could improve the flavor, taste usability for processing, and higher quality [35]. Mroczek-Sosnowska et al [51] reported that 24 hours after the slaughter of broilers that, when still eggs, were injected with colloidal CuSO_4_ as nanoparticles, their breast and leg muscles showed a pH decrease and a WHC increase. The CL of the breast and thigh of chickens fed diets supplemented with HME-Cu or IN-Cu increased compared to the control. Although it is unclear why supplemental Cu affected the CL, the result was related to the pH decline. L* acts as an indicator of PSE (pale, soft, and exudative condition) meat. This study showed that the L* value was increased with the Cu and HME-Cu supplementation compared to the control and the IN-Cu supplementation. Furthermore, the L* value was increased with a low pH; and in the control and the IN-Cu-supplemented diets that resulted in high L* values, the meat color appeared as dark compared to that with the HME-Cu supplementation, which coincided with the findings of the study of Sifa et al [30].

A previous study reported that nano-Cu had a reduced size and an increased surface area, which improved the bioavailability of Cu than in the inorganic form [37]. The Cu excreta increased with the Cu supplementation, except for the source in the study. In addition, HME-Cu increased the Cu content of the liver compared to the control and reduced the Cu content of the feces compared to the IN-Cu-supplemented diets. Our previous studies reported that HME improved the bioavailability and metabolic function of minerals, which in turn decreased the fecal mineral output in weanling pigs and broiler chickens [23–28]. Similarly, in chickens, diet supplementation with 20 mg/kg of inorganic and organic Cu increased the Cu content of their liver; and in weanling pigs, diet supplementation with 50 mg/kg of nano-Cu reduced their fecal Cu content compared to diet supplementation with 50 mg/kg of inorganic Cu [41,52].

In conclusion, this study showed that supplementation of broiler diets with HME-Cu at the requirement level (16 mg/kg) did not affect the growth performance and physiological function of Cu. However, HME-Cu improved the meat quality and the bioavailability of Cu. The effect of pharmacological doses of HME-Cu on broiler chickens must be further investigated.

## Figures and Tables

**Table 1:** Formula and chemical composition of experimental diets (as-fed basis)

Items	Phase 1 (0 to 14 d)	Phase 2 (14 to 35 d)
Ingredients (%)		
Corn (9%)	54.16	60.82
Soybean meal (48%)	32.63	24.31
Corn gluten meal (64%)	4.00	5.00
Soy-oil	4.47	4.30
Choline chloride (50%)	0.05	0.05
Mono-dicalcium phosphate	1.55	1.48
Limestone	1.54	1.50
Salt	0.20	0.20
Mineral premix^1)^	0.15	0.15
Vitamin premix^2)^	0.15	0.15
L-Lysine (24%)	-	1.42
DL-Threonine (99%)	-	0.22
DL-Methionine (99%)	0.18	0.37
DL-Tryptophan (10%)	0.92	0.03
Total	100.00	100.00
Calculated nutrient value		
Metabolizable energy (kcal/kg)	3,050	3,150
Crude protein (%)	22.00	20.50
Calcium (%)	0.93	0.84
Available phosphorus (%)	0.45	0.42
SID lysine (%)	1.24	1.11
SID methionine+cystine (%)	0.92	0.85
Analyzed nutrient value		
Crude protein (%)	22.35	20.43
Crude fat (%)	7.43	8.12
Crude ash (%)	5.43	5.35

SID, Standardized ileal digestible.

1) Supplied per kilogram of diet: 20 mg iron; 0.25 mg cobalt; 110 mg zinc; 120 mg manganese; 1.25 mg iodine; 0.30 mg selenium.

2) Supplied per kilogram of diet: 10,000 IU vitamin A; 4,500 IU vitamin D_3_; 65 mg vitamin E; 3.2 mg vitamin K_3_; 3.2 mg vitamin B_1_; 9 mg vitamin B_2_; 6 mg vitamin B_6_; 0.02 mg vitamin B_12_; 17 mg pantothenic acid; 60 mg niacin; 0.30 mg biotin; 2.30 mg folic acid.

**Table 2:** Calculated and analyzed dietary Cu levels in broilers

Cu sources^1)^	Calculated Cu content (mg/kg)	Analyzed Cu content (mg/kg)^2)^
Control diet	0	5.61
IN-Cu	16	22.21
HME-Cu	16	23.03

1) Control, without supplemental Cu; IN-Cu, CuSO4; HME-Cu, hot-melt extruded CuSO_4_.

2) Analyzed Cu values are based on chemical analysis of triplicate samples of each diet.

**Table 3:** Effect of dietary Cu concentrations and sources on growth performance in broilers

Items	Control^1)^	IN-Cu^1)^	HME-Cu^1)^	SEM	p-value
Phase 1 (1 to 14 d)					
Weight gain (g/bird)	407	402	410	4.76	0.496
Feed intake (g/bird)	565	573	567	10.39	0.763
G:F	0.72	0.71	0.72	0.03	0.940
Phase 2 (15 to 35 d)					
Weight gain (g/bird)	1,681	1,713	1,719	8.09	0.079
Feed intake (g/bird)	2,869	2,906	2,892	14.29	0.318
G:F	0.59	0.59	0.59	0.01	0.801
Overall (0 to 35 d)					
Weight gain (g/bird)	2,087	2,115	2,129	8.79	0.078
Feed intake (g/bird)	3,435	3,460	3,479	20.14	0.386
G:F	0.61	0.61	0.62	0.01	0.806

SEM, standard error of means; G:F, gain to feed ratio (g gain/feed).

1) Control, without supplemental Cu; IN-Cu, CuSO_4_; HME-Cu, hot-melt extruded CuSO_4_.

**Table 4:** Effect of dietary Cu concentrations and sources on digestibility of nutrients in broilers

Items	Control^1)^	IN-Cu^1)^	HME-Cu^1)^	SEM	p-value
Phase 1 (d 14)					
DM	72.41	73.17	72.31	0.49	0.243
GE	72.45	72.98	73.69	0.46	0.117
CP	64.31	64.95	65.30	0.38	0.175
Cu	35.78	36.03	41.01	0.98	0.066
Phase 2 (d 35)					
DM	71.23	70.57	71.52	0.92	0.494
GE	72.97	73.28	73.91	0.86	0.467
CP	64.09	63.39	63.62	1.16	0.686
Cu	34.38^b^	35.27^b^	40.47^a^	0.92	0.014

SEM, standard error of means; DM, dry matter; GE, gross energy; CP, crude protein.

1) Control, without supplemental Cu; IN-Cu, CuSO_4_; HME-Cu, hot-melt extruded CuSO_4_.

a, b Means within a row with different superscripts differ significantly (p<0.05).

**Table 5:** Effect of dietary Cu concentrations and sources on gut microbiota in broilers

Items	Control^1)^	IN-Cu^1)^	HME-Cu^1)^	SEM	p-value
Small intestine					
Total anaerobic bacteria	8.57	8.48	8.42	0.08	0.228
Lactobacillus spp.	7.16	7.27	7.37	0.11	0.215
Clostridium spp.	5.66	5.54	5.62	0.06	0.248
* Escherichia coli*	6.55	6.63	6.59	0.05	0.362
Cecum					
Total anaerobic bacteria	9.13	9.18	9.22	0.04	0.227
Lactobacillus spp.	8.48	8.58	8.61	0.07	0.271
Clostridium spp.	6.34	6.28	6.46	0.13	0.374
* Escherichia coli*	7.25	7.15	7.19	0.04	0.099

SEM, standard error of means.

1) Control, without supplemental Cu; IN-Cu, CuSO_4_; HME-Cu, hot-melt extruded CuSO_4_.

**Table 6:** Effect of dietary Cu concentrations and sources on small intestinal morphology in broilers

Items	Control^1)^	IN-Cu^1)^	HME-Cu^1)^	SEM	p-value
Duodenum					
VH	1,144	1,167	1,148	22.33	0.465
CD	191	204	199	9.23	0.306
VH:CD	6.08	5.86	5.89	0.25	0.537
Jejunum					
VH	808	813	835	21.62	0.398
CD	171	161	164	4.87	0.160
VH:CD	4.79	5.06	5.11	0.17	0.222
Ileum					
VH	519	526	528	13.27	0.625
CD	142	143	147	3.27	0.414
VH:CD	3.69	3.69	3.61	0.11	0.618

SEM, standard error of means; VH, villus height; CD, crypt depth; VH:CD, ratio of villus height to crypt depth ratio.

1) Control, without supplemental Cu; IN-Cu, CuSO_4_; HME-Cu, hot-melt extruded CuSO_4_.

**Table 8:** Effect of dietary Cu concentrations and sources on Cu bioavailability in broilers

Items	Control	IN-Cu	HME-Cu	SEM	p-value
Phase 1 (d 14)					
Plasma (mg/L)	0.15	0.18	0.16	0.02	0.489
Excreta (mg/kg)	13.25^b^	39.43^a^	33.14^a^	1.73	<0.001
Phase 2 (d 35)					
Plasma (mg/L)	0.15	0.17	0.18	0.02	0.540
Liver (mg/kg)	12.50^b^	16.82^ab^	17.97^a^	0.91	0.031
Excreta (mg/kg)	32.08^c^	109.79^a^	103.66^b^	1.13	<0.001

Control, without supplemental Cu; IN-Cu, CuSO_4_; HME-Cu, hot-melt extruded CuSO_4_; SEM, standard error of means.

a–c Means within a row with different superscripts differ significantly (p<0.05).

**Table 7:** Effect of dietary Cu concentrations and sources on meat quality in broilers

Items	Control^1)^	IN-Cu^1)^	HME-Cu^1)^	SEM	p-value
Breast					
pH_24 h_	6.14^a^	5.97^b^	5.80^c^	0.03	0.001
CL (%)	23.65^c^	26.54^b^	29.03^a^	0.33	<0.001
WHC (%)	51.99^c^	53.66^b^	59.76^a^	0.30	<0.001
L* (lightness)	46.30^c^	48.34^b^	50.26^a^	0.32	<0.001
a* (redness)	7.07	6.72	7.17	0.29	0.539
b* (yellowness)	9.51	9.70	9.77	0.33	0.752
Thigh					
pH_24 h_	6.29^a^	6.14^b^	6.00^c^	0.03	<0.001
CL (%)	31.85^b^	32.90^ab^	34.21^a^	0.29	0.007
WHC (%)	52.85^b^	55.41^ab^	56.55^a^	0.52	0.013
L* (lightness)	48.75^b^	49.74^ab^	51.33^a^	0.34	0.010
a* (redness)	4.31	4.93	5.22	0.19	0.076
b* (yellowness)	7.07	6.66	6.79	0.34	0.632

SEM, standard error of means; CL, cooking loss; WHC, water-holding capacity.

1) Control, without supplemental Cu; IN-Cu, CuSO_4_; HME-Cu, hot-melt extruded CuSO_4_.

a–c Means within a row with different superscripts differ significantly (p<0.05).
